# Narrowing down the region responsible for 1q23.3q24.1 microdeletion by identifying the smallest deletion

**DOI:** 10.1038/s41439-019-0079-1

**Published:** 2019-10-18

**Authors:** Takao Hoshina, Toshiyuki Seto, Taro Shimono, Hiroaki Sakamoto, Torayuki Okuyama, Takashi Hamazaki, Toshiyuki Yamamoto

**Affiliations:** 1 Department of Pediatrics, Osaka City University Graduate School of Medicine, Osaka, Japan; 2 Department of Medical Genetics, Osaka City University Graduate School of Medicine, Osaka, Japan; 3 Department of Radiology, Osaka City University Graduate School of Medicine, Osaka, Japan; 4 Department of Neurosurgery, Osaka City University Graduate School of Medicine, Osaka, Japan; 5 Department of Clinical Laboratory Medicine/Center for Lysosomal Storage Diseases, National Center for Child Health and Development, Tokyo, Japan; 6 Tokyo Women’s Medical University Institute for Integrated Medical Sciences, Tokyo, Japan; 7 Institute of Medical Genetics, Tokyo Women’s Medical University, Tokyo, Japan

**Keywords:** Medical research, Genetics

## Abstract

Interstitial deletions of 1q23.3q24.1 are rare. Here, chromosomal microarray testing identified a de novo microdeletion of arr[GRCh37]1q23.3q24.1(164816055_165696996) × 1 in a patient with moderate developmental delay, hearing loss, cryptorchidism, and other distinctive features. The clinical features were common to those previously reported in patients with overlapping deletions. The patient’s deletion size was 881 kb—the smallest yet reported. This therefore narrowed down the deletion responsible for the common clinical features. The deleted region included seven genes; deletion of *LMX1A*, *RXRG*, and *ALDH9A1* may have caused our patient’s neurodevelopmental delay.

## Introduction

Interstitial microdeletions neighboring the 1q24 region are rare, and approximately 10 patients with such deletions have been reported to date^1,2^. One genotype–phenotype correlation study suggested that several regions may be responsible for the phenotypic features. Chatron et al.^1^ reported that patients with 1q24q25 microdeletions have distinctive phenotypes, including growth deficiency; they suggested that a 1.9-Mb region at 1q24.3q25.1 was the shortest region of overlap (SRO). Moreover, they proposed that deletions of two of the genes within the SRO, namely, the dynamin 3 gene (*DNM3*) and the centromere protein L gene (*CENPL*), were potential causes of intellectual disability and growth deficiency, respectively. However, some patients have microdeletions in the region proximal to the 1q24q25 region^1^.

Here, we report the smallest microdeletion yet found in the 1q23q24 region, and gene deletions that may be related to clinical features such as moderate developmental delay, hearing loss, and cryptorchidism, in a young boy.

**Fig. 1:**
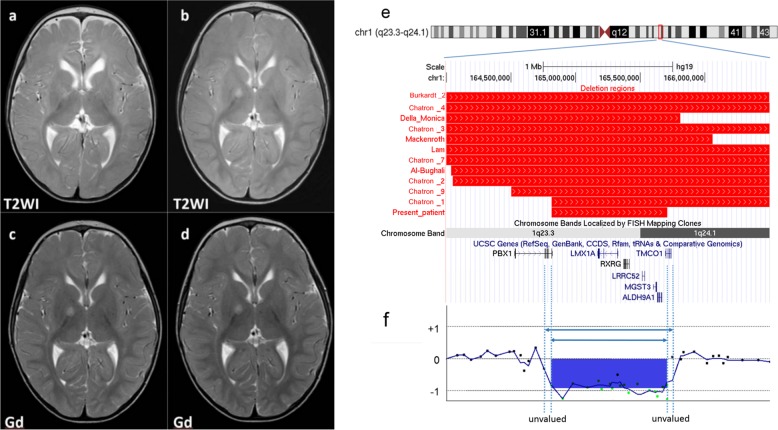
Results of brain magnetic resonance imaging and genome mapping around the 1q23.3q24.1 region. Axial T2-weighted images were taken at **a**, **c** 12 months and **b**, **d** 19 months. **c**, **d** A T2 high-intensity area found in the right genu of the internal capsule was not enhanced by gadolinium administration. **e** The deleted regions in our patient and in patients reported previously are shown by red bars. **f** Results of chromosomal microarray analysis of our patient

The patient was a boy aged 2 years and 8 months who had been born at full term with a birth weight of 2818 g and a normal spontaneous delivery. The pregnancy was uneventful. His parents were not consanguineous. Soon after birth, congenital hearing loss and cryptorchidism were found. At 4 months, poor weight gain and developmental delay were noted. At 10 months, the boy was referred to our hospital for further examination; at this time, he could turn over but could not sit unsupported. He had distinctive features, including mild hypotelorism, low-set ear, a tented upper lip, and a small jaw. Neurological examination revealed axial hypotonia. There were no abnormalities on routine laboratory examination, including plasma amino acids and urinary organic acids. Chromosomal G-banding showed a normal male karyotype of 46,XY. Brain magnetic resonance imaging (MRI) at 19 months revealed T2 high intensity in the right genu of the internal capsule (Fig. 1b, d); this had not been noted at 12 months (Fig. 1a, c). This area was not enhanced by gadolinium administration (Fig. 1c, d). Serum tumor markers were negative, with alpha-fetoprotein 8.0 ng/mL and human chorionic gonadotropin beta < 0.1 ng/mL.

At the time of writing, the boy was 82.0 cm tall ( <third percentile); he weighed 9.9 kg ( <third percentile) and had an occipitofrontal circumference of 48 cm (10th to 25th percentile), indicating growth impairment but not microcephaly. The boy can walk with support but has no meaningful vocabulary, indicating moderate developmental delay.

This study was approved by the ethical committees of Osaka City University and Tokyo Women’s Medical University. After obtaining written informed consent from the patient’s family, we performed chromosomal microarray testing using the Agilent microarray 60 K (Agilent Technologies, Santa Clara, CA, USA), as described previously^3^. A genomic copy number loss was identified at the chromosome region of 1q23.3q24.1 with minimum and maximum extents of 164,816,055–165,696,996 (881-kb) and 164,761,881–165,739,928 (978-kb), respectively [GRCh37/hg19]. (Fig. 1e, f). The same testing of both parents revealed that they did not have this deletion, indicating its de novo origin.

We identified a 1q23.3q24.1 microdeletion in a patient with developmental delay, growth impairment, hearing loss, and cryptorchidism. In the literature, there are reports of at least 10 patients who have shown deletions overlapping with that in our patient^1,2,4–7^. The clinical features of these patients are summarized in Table 1. Developmental delay, intellectual disability, or both were observed in all patients. Growth impairment, hypotonia, and distinctive features were also noted in most of the patients (Table 1). Depiction of the deletion regions of these patients on a genome map shows that the deletion identified in our patient is the smallest yet found (Fig. 1e). The genomic coordinates of the copy number variation in our patient delimit the maximum extent (Fig. 1f). In contrast, in the comparison cases reported by Chatron et al.^1^, the coordinates delimit the minimum extent, and in the patient reported by Al-Bughaili^4^, they were exact.

**Table 1:** Clinical features of our patient and patients from previous studies

Patient	Our patient	Al–Bughaili et al.^4^	Mackenroth et al.^5^	Lam et al.^6^	Chatron et al.^1^						Burkardt et al.^2^	Della Monica et al.^7^	
					P1	P2	P3	P4	P7	P9	P2		
Chromosomal region	1q23.3q24.1	1q23.3q24.2	1q23.3q24.1	1q23.3q25.1	1q23.3q25.1	1q23.3q25.2	1q23.3q25.1	1q23.3q24.1	1q23.3q25.1	1q23.3q24.3	1q23.3q25.1	1q23.3q24.2	
Coordinates (hg19)													
Start	164,816,055	164,031,508	163,193,466	163,255,872	164,816,055	164,048,582	161,650,414	160,797,550	163,716,255	164,501,003	163,760,542	162,573,376	
End	165,696,996	170,654,599	166,058,476	174,607,307	173,490,508	177,852,133	174,926,542	167,022,133	174,427,602	171,424,595	172,532,315	167,543,376	
Size of deletion	880,941	6,623,091	2,865,010	11,351,435	8,674,453	13,803,551	13,276,128	6,224,583	10,711,347	6,923,592	8,771,773	4,970,000	
Age	2 years	5 years	5 years	9 years	7 years	5 years	2 days	10 years	1 year	5 years	5 years	5 years	
Sex	Male	Male	Female	Male	Male	Female	Female	Male	Female	Female	Female	Male	Male 6 Female 6 (*n* = 12)
Growth													
IUGR	–	–	−	+	+	+	+	+	+	Ns	+	Ns	7/10 (70%)
Postnatal growth impairment	+	+	–	+	+	+	–	+	+	+	+	–	9/12 (75%)
Microcephaly	–	+	–	+	+	+	+	–	+	+	+	–	9/12 (75%)
Delayed bone age	NA	NA	NA	+	NA	NA	NA	NA	+	–	+	+	4/5 (80%)
Neurodevelopmental													
Feeding difficulties	+	NA	–	+	–	+	NA	+	+	–	Ns	–	5/9 (56%)
Developmental delay/intellectual disability	+	+	+	+	+	+	NA	+	+	+	+	+	11/11 (100%)
Walking delay	+	+	+	+	–	+	NA	+	–	+	NA	NA	7/9 (78%)
Speech delay	+	+	+	+	+	+	NA	+	–	+	+	+	10/11 (91%)
Hypotonia	+	NA	–	+	+	+	+	–	+	–	+	+	8/11 (73%)
Seizures	–	NA	–	–	–	+	–	–	–	–	NA	–	1/10 (10%)
Hearing loss	+	NA	–	+	–	–	–	–	–	+	NA	–	3/10 (30%)
Facial dysmorphism													
Sparse hair/eyebrows	–	–	–	+	+	–	–	–	–	+	–	–	3/12 (25%)
Hypertelorism	–	+	–	–	–	+	+	–	–	–	–	–	3/12 (25%)
Ear anomalies	+	+	+	+	+	+	+	+	+	+	+	+	12/12 (100%)
Bulbous/broad nose	+	+	+	+	+	+	+	+	+	–	–	–	9/12 (75%)
Abnormal lip/palate	–	–	–	–	+	–	–	–	+	–	+	–	3/12 (25%)
Micro/retrognathia	+	+	+	+	+	+	+	–	+	–	+	+	10/12 (83%)
Short/broad neck	–	–	–	+	–	+	–	–	+	–	+	–	4/12 (33%)
Hands/feet													
Small hands/feet	–	+	–	+	–	–	+	–	+	+	–	–	5/12 (42%)
Brachydactyly	–	+	–	+	–	+	+	–	+	+	–	–	6/12 (50%)
Fifth-finger clinodactyly	–	+	–	+	+	+	+	–	+	+	+	+	9/12 (75%)
Broad thumb	–	+	–	+	+	–	+	–	–	–	+	–	4/12 (33%)
Single palmar crease	–	NA	–	+	+	–	+	–	+	–	+	–	5/11 (45%)
Malformations													
Cryptorchidism	+	+	–	+	+	–	–	+	–	–	NA	–	4/11 (36%)
Kidney	–	+	+	+	–	+	+	+	–	+	NA	+	8/11 (73%)
Heart	–	–	+	–	+	+	+	–	–	+	NA	–	5/11 (45%)
Brain	–	+	NA	+	–	+	–	–	–	–	NA	+	4/10 (40%)
Skeleton	–	+	–	+	–	–	–	+	+	NA	+	–	5/11 (45%)

*IUGR* intrauterine growth retardation, *NA* not available, *Ns* not specified, *P* patient

Genomic regions for patients reported by Della Monica et al. (2007) were transferred to hg19

Among the genes included in the region deleted in our patient (Fig. 1e), seven are included in the Online Mendelian Inheritance in Man database (OMIM; https://omim.org/), and mutations or deletions of two—namely, the pre-B-cell leukemia homeobox 1 gene (*PBX1*; MIM #176310) and the transmembrane and coiled-coil domains 1 gene (*TMCO1*; MIM #614123)—have been associated with diseases (see Supplementary information). *PBX1* is one of the three-amino-acid loop extension (TALE) homeodomain-containing transcription factors and is involved in early development in mammals^8^. Mutations or deletions of *PBX1* are responsible for congenital anomalies of the kidney and urinary tract^9^. Although our patient had no renal abnormalities, eight other patients had renal malformation^1,5–7^ (Table 1). This would be derived from incomplete penetrance of phenotypic features. Mackenroth et al.^5^ proposed disruption of *PBX1* as a potential cause of renal malformation, albeit perhaps with incomplete penetrance, as there was no renal malformation in patient 7 in the series of Chatron et al.^1^.

*TMCO1* is a member of the DUF841 superfamily of several eukaryotic proteins with unknown function^10^. Xin et al.^10^ reported that a homozygous frameshift mutation in *TMCO1* causes craniofacial dysmorphism, skeletal anomalies, and developmental delay. Because these clinical conditions are associated with autosomal recessive traits^9^, this gene would not be related to the clinical features of our patient.

The LIM homeobox transcription factor 1A gene (*LMX1A*) is essential for neuronal differentiation into dopaminergic neurons in the midbrain^11^, and the retinoic acid receptor RXR-gamma isoform C gene (*RXRG*), expressed mainly in the striatum, encodes a nuclear retinoic acid receptor^12^. Mackenroth et al.^5^ proposed that disruption of these genes was a cause of developmental delay. In addition, the SRO includes the gene encoding aldehyde dehydrogenase 9 family member A1 (*ALDH9A1*), which catalyzes the dehydrogenation of gamma-aminobutyraldehyde to gamma-aminobutyric acid. Bartlett et al.^13^ suggested that *ALDH9A1* disruption may be related to autism, so the gene may also be associated with brain development. The analysis of more patients will be needed for a precise genotype–phenotype correlation study.

Our patient incidentally had an abnormal MRI finding in the basal ganglia that had not been apparent earlier. To rule out a malignant tumor, a detailed examination was performed, and a spot with high T2 intensity was found that was not enhanced by gadolinium. We were unable to reach a final conclusion about the significance of this lesion. Continuous follow-up of this finding will be needed.

## Supplementary information

Supplementary Information

## Data Availability

The relevant data from this Data Report are hosted at the Human Genome Variation Database at 10.6084/m9.figshare.hgv.2627 10.6084/m9.figshare.hgv.2630 10.6084/m9.figshare.hgv.2633 10.6084/m9.figshare.hgv.2636 10.6084/m9.figshare.hgv.2639 10.6084/m9.figshare.hgv.2642 10.6084/m9.figshare.hgv.2645
